# Exploring the Intersections of Migration, Gender, and Sexual Health with Indonesian Women in Perth, Western Australia

**DOI:** 10.3390/ijerph192013707

**Published:** 2022-10-21

**Authors:** Corie Gray, Gemma Crawford, Bruce Maycock, Roanna Lobo

**Affiliations:** 1 Collaboration for Evidence, Research and Impact in Public Health, School of Population Health, Curtin University, Bentley 6102, Australia; 2 College of Medicine & Health, University of Exeter, Exeter EX4 4PY, UK

**Keywords:** migrant, intersectionality, sexual health, gender

## Abstract

This paper explores the intersections of migration, gender, and sexual health with Indonesian women living in Perth, Western Australia. The study was part of a larger participatory action research project to co-design an intervention to increase HIV testing in migrant Indonesian women. Unstructured interviews were conducted with adult Indonesian women (*n* = 10) on their experiences of migration and sexual health. Zimmerman’s migration phases (pre-departure and travel, destination, and return) provided a framework to conceptualize women’s migration journeys. We found that women’s migration journeys were shaped by gender, with male-led migration often reinforcing gender norms. Structural and sociocultural factors (including visa status) influenced women’s sense of belonging while living in Australia, such as help-seeking behaviour and engagement in sexual relationships. Intersecting factors of gender, culture, and the migration process should be considered when designing public health interventions to improve women’s sexual health, in particular migration policies and procedures.

## 1. Introduction

An individual’s migration experience is characterized by unique phases pre-, during and post-migration, and upon their return. Zimmerman et al. (2011) describe the migration process as a complex, multistage cycle that can be entered multiple times and argue that public health interventions must target each stage of the cycle [1]. Limited studies have explored the impact of health across all migration phases [1,2] and few public health interventions show consideration across migration phases [3].

Addressing migrant health inequalities remains a global challenge [4,5]. For the most part, an understanding of migrant health is mostly derived from the concept of the ‘healthy migrant effect’, in which migrants tend to be healthier than the host population, attributed to the selective process of migration (i.e., only healthy people successfully migrate) [6]. However, studies suggest that migrant health tends to decline over time and is comparatively worse than newly arrived migrants [7]. For example, an Australian longitudinal study reported that migrants who have been in Australia for more than 10 years had worse mental and self-assessed health than Australian-born individuals [8,9]. One explanation for the erosion of health amongst migrants in host countries is the experience of a combination of cultural, legal, and social barriers to health and social protections which may negatively influence health outcomes for migrants. Understanding of health and health beliefs may also shift during migration [10]. Consequently, migration may be considered a social determinant of health [11,12] which may also influence other social determinants of health, such as social inclusion, income, and working conditions [6]. Gender has been reported to compound health inequalities for migrants, with some evidence that the health of migrant women is worse than men, despite leading healthier lifestyles than men [13,14]. Researchers have suggested that this may be due to migration factors that favor men, in addition to gender inequalities experienced both pre- and post-migration [13].

An intersectionality framework offers additional insights about the multifactorial impact of determinants on migrant health both pre-, during, and post-migration [6,15,16]. This includes the multiplication of marginalization, acknowledging that a single-axis understanding of gender or race alone obscures multiple dimensions of discrimination [6]. Some scholars have criticized early theories around migration, including the ‘healthy migrant effect’, as being ‘genderblind’ [17]. When stratifying by gender, studies suggest differences in health literacy, health service access, and health outcomes, with women fairing worse than men [13,16,18,19]. Health differences between men and women are in part due to gender inequalities experienced both in the country of origin and the host country; typically affecting migrant women worse than native-born women [16]. Despite recognition of the impact of gender and migration on health [16,20], few studies have explored how migration [1] influences, and is influenced by, gender and its impact on health [2,20]. 

Like gender, sexual health is a social construct, influenced by social, cultural, political, and economic factors [21,22] including gender relations, norms, and expectations [16]. Australian evidence suggests sexual health inequalities are experienced by women, also shaped by pre- and post-migration factors [21]. Migration may change gender relations, affecting women in both their host country and country of origin [20]. For example, migrant women are less likely to access contraceptives and health services and are at greater risk of acquiring sexually transmitted infections (STI) or HIV [23,24,25]. There are few studies that explore how sexuality and sexual health are influenced by migration [26,27]. Much of the research has focused on access to healthcare generally; fewer studies have considered how migration impacts health more broadly, including the factors that influence health, such as gender [2,17,20,27,28].

In Australia, peak organisations for sexual health have advocated for a more nuanced understanding of how migration and gender and cultural norms shape sexual health practices [29]. Sexual health interventions developed for migrants in Australia have tended to apply a ‘one size fits all’ approach, as reported in a review by Crawford and colleagues [30]. There are calls for the development of more tailored interventions for specific migrant populations [3,31,32,33], including in national policy documents [34]. Developing appropriate health promotion interventions requires a greater understanding of how sexual health is influenced by broader sociocultural factors [22,35]. However, there exists a dearth of evidence for sexual health promotion practitioners to draw on, particularly in relation to migrant women [2]. Available information usually clusters migrants into fairly homogenous groups, for example, ‘Asian’ or ‘Southeast Asian’ migrants, and fails to take into consideration significant differences in language, ethnicity, culture, class, and religion (as a few examples) [2]. Within countries of birth, women have considerably different backgrounds, including reasons for migration and migration experience, and research focused on specific ethnic groups or countries of birth is required to inform targeted interventions.

This research was part of a larger study, known as Srikandi (an Indonesian folklore) to co-design a sexual health intervention with Indonesian women living in Perth [36]. People from Indonesia constitute 1.2% of international migrants in Australia [37], however, there is limited research on the health and health outcomes of Indonesian migrants and no known studies on sexual health [2,3,35]. This research aimed to explore the intersection of migration, gender, and sexual health among Indonesian women living in Perth, Western Australia.

## 2. Methods

The broader Srikandi multi-method participatory action research (PAR) project is composed of a systematic review, a content analysis, focus groups, and co-design workshops, reported elsewhere [2,33]. PAR uses a process of reflection, influenced by an understanding of the local context, and should lead to participants experiencing increased control over their lives [38]. PAR emphasizes involvement of the community, in which data collection is undertaken to inform action to address health inequalities [38].

Interviews were selected to complement other project components, consistent with the flexibility and reflexivity of PAR. Interviews are a commonly used PAR method, and this research provided rich insights regarding individuals’ experiences of migration, particularly around gender, sexual health, and risk. We conducted interviews with 10 Indonesian women living in Perth, WA using reflexive thematic analysis (TA). Reflexive TA supports the subjective nature of qualitative research and is often conducted by a sole researcher through an organic coding process [39,40].

### 2.1. Research Team

This research was undertaken by an Australian-born female doctoral student supervised by three university-based academics (two females and one male). All interviews were undertaken by the first author, who had limited knowledge of Indonesia or the Indonesian community living in Perth prior to the commencement of the project and was considered a ‘cultural outsider’ [41]. The study recruited six Indonesian migrant women living in Perth, Western Australia, as community researchers (CRs). Further details of the research team, including community researchers, are detailed elsewhere [36].

### 2.2. Participants

Ten women participated in interviews (none were CRs). Women were born in Indonesia and over the age of 18 and living in metropolitan Perth, WA, and were able to take part in an English language interview. Interviews involved women who had been involved in previously conducted focus groups (*n* = 8) and those who were reluctant to participate in a group setting. Recruitment into focus groups and interviews occurred through social media (i.e., WhatsApp and Facebook) and email lists, and word of mouth via community researchers. Selected focus group participants were then invited to take part in the interviews, based on the diversity of their experiences described during the focus group and their ability to take part in an English-only interview. Women were approached solely by the first author. To protect interviewees’ confidentiality during the data analysis, in all but one instance, community researchers did not know who had or had not participated in interviews. One participant asked a community researcher to attend the interview to provide language support. Interviews were concluded when we felt we had sufficient variation in women’s broad migration experiences; we did not believe it possible to reach data saturation, consistent with the reflexive TA procedure [39,40].

### 2.3. Participant Characteristics

Women had mostly arrived more recently to Australia (*n* = 6, arrived 2015–2018) and were predominately on student visas (*n* = 5). Most women (*n* = 7) were married and with children. Four participants had previously undergone testing for HIV. Full demographic characteristics are presented in Table 1 for nine participants, as one participant did not consent to provide demographic data. 

### 2.4. Procedure

This study was approved by Curtin University Human Research Ethics Committee (approval number HRE2018-0790). The three domains of the consolidated criteria for reporting qualitative research (COREQ) informed the study design [42]. Domains of the investigation were based on gaps identified in previously conducted focus groups and through consultation with community researchers. Interviews were unstructured and exploratory in nature. Broadly, questions focused on women’s experiences of arriving and living in Australia and questions about women’s intimate relationships and sexual health. 

Participants were provided with an information sheet, summarised to them verbally. Women were encouraged to ask questions before providing written consent. Demographic data were collected, and participants offered information about local sexual health services. Interviews were unstructured and ranged from 2–3.5 h (excluding breaks for food, bathroom, or prayer) and were digitally recorded. Participants chose a quiet public location to complete the interview. To enhance rapport, the interviewer purchased a beverage for participants and conversed with them for about 15–20 min prior to commencing the interview.

### 2.5. Data Analysis

The first author undertook all data analysis including checking of transcripts and initial coding to maintain participants’ confidentiality (i.e., no other research team members viewed data). This process enabled the first author to become fully immersed in the data, facilitating a deeper understanding, and increasing credibility, trustworthiness, and rigour [43,44]. To protect the confidentiality of participants, quotes and description of certain experiences have not been included in this publication.

Interviews were transcribed verbatim by either the first author or a professional transcriber, unknown to any of the research participants. Transcripts were checked by the first author by reviewing the audio and reading the written text to ensure accuracy. The analysis did not begin until all data had been collected. In the first instance, transcripts were coded using NVivo (QSR International Pty Ltd. Victoria, Australia) [45] using an inductive approach. Here the first author developed summaries of the women’s stories, focusing on the experiences described and supported by large, contextual quotes (i.e., chunking) [46]. Points of further investigation were noted and initial thoughts were documented. This process allowed the researcher to easily recall different experiences and provided a mechanism to identify similarities and differences between interviews. De-identified narratives were discussed with the academic team and community researchers, providing the opportunity for further reflection and increasing credibility and trustworthiness [43,44]. Narratives were considered through Zimmerman et al.’s (2011) phases of migration, a framework used to identify points in time for public health interventions [1]. The narratives and codes were reviewed and grouped into themes and sub-themes where appropriate. Written themes were shared with the academic team, and further refined by the first author with feedback.

## 3. Results

**Figure 1:**
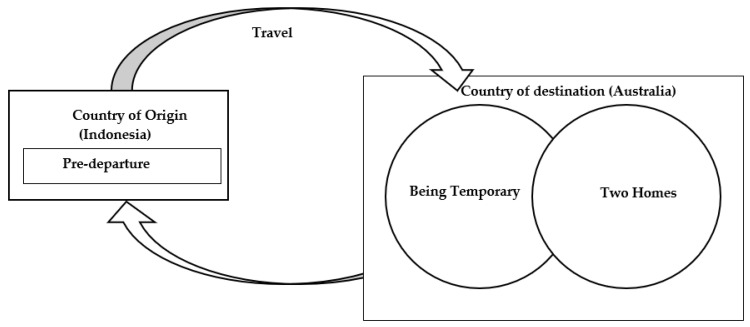
Thematic map.

Zimmerman’s phases of migration—pre-departure and travel, destination, and return were adapted to conceptualise themes related to women’s experiences of sexual health (Figure 1). Women described their sexual health knowledge and attitudes, and the context of migration during the pre-departure and travel phase. Two common and overlapping themes were identified during the destination phase: feeling like a temporary migrant or feeling at home in two countries. An overview of themes and sub-themes are included (Table 2).

### 3.1. Pre-Departure and Travel: Motivations, Support, and Gender Roles

Women’s reasons for migration varied. In many cases, migration was influenced by gender roles. Women’s travel to Perth was undertaken in the context of little sexual health education and with expectations that men were ‘sexually naïve’.

#### 3.1.1. ‘I’m a Little Curious’: Expectations of Marriage, Sexual Naivety, and Self-Taught Sexual Education

Women described Indonesia as “a patriarchal society”, in which men were emphasised as the main financial contributors. Women emphasised the expectation that women “should respect their husband”, reinforced both in their culture and through religion. Women’s noted that their culture valued women’s ability “to marry off… and have grandchildren”—subsequently, there was less emphasis on women’s education or employment opportunities compared to men. In many regards, women noted that women were considered “male’s property”, in which men had some control over women’s livelihoods (e.g., finances and decisions on where to live). While there was a view that this was changing, with women being afforded more opportunities for employment and education even after marriage, there still existed strong social pressure to marry. Women described having to live “a sheltered life” in Indonesia, based on status as a woman, that had restricted expression of sexuality:

“*In Indonesia, it’s like a culture when a girl—just better to stay at home. If you’re a boy, then you can go. I mean we (girls) have to like, you know, be more protected.*”

—Student visa, 2019

Related, there was an importance placed on men’s sexual pleasure that was not afforded to women. Participants described a culture that valued women’s virginity, and where women’s sexuality was required to be self-policed (i.e., women were not allowed to expose themselves to information about sexual intercourse). Women were expected to be sexually naïve, with limited knowledge of anything relating to sex, and dependent on their husbands to teach them. In Indonesia, all women described a lack of sexual health education and the taboo nature of sex. School-based sexual education emphasised abstinence and used fear-based tactics regarding STIs and pregnancy. Subsequently, women’s understanding of sexual intercourse and sexuality came from the media, where women were often shown to be submissive, and there was limited reference to safer sex practices, as described by one participant:

“*You can only learn it (sexual health) from your friends and internet… you know, boys would bring the porn magazine. And then the comic, the porn—soft porn comic book and then they share it to themselves and then even though you are a female, you kinda like come across to them and will be like, “Oh, can I see that as well?” And you kinda,—you know, learn from them (porn magazines)*”

—Permanent resident, 2007

Despite social expectations that women were to be sexually naïve, several women admitted to being interested in sex prior to marriage and had engaged in sexual activities. Receiving little education from formal institutions such as government or schools, and being reluctant to discuss with social networks, women relied on media to explore their sexuality. This was predominately through pornography (either magazines or videos), sexualised comics, and particularly through the 50 Shades of Grey series (a popular book and movie series). Women accessed this media either online or via men. Accessing this material online required a VPN (virtual private network) whilst in Indonesia. As a result, a number of women shared their experiences of being diagnosed with an STI or an unplanned pregnancy prior to migrating, citing a lack of knowledge about sexual health pre-migration.

#### 3.1.2. ‘You Already Know the Culture’: Maintaining Gender Roles in Migration

For women who migrated to the community, or with family, gender roles were often maintained, or at times, further reinforced. For most women, the decision to migrate was not their own and instead occurred as part of familial duty. Women typically migrated to support their husbands’ aspirations to seek further education or employment in Perth. This migration was often at the expense of their own careers or lifestyles, reinforcing gender roles of males as main income earners and women as caretakers. For example, Participant 8 migrated to Perth at the request of her husband, who had moved the year before to undertake his PhD. He struggled to cook and do household chores, so asked her to relocate so she could manage the house, giving up her career in the process. Moving to Perth reinforced the traditional gender role of women as carers and reduced her employment opportunities:

“*He came here and started PhD.… He’s not quite good in cooking. He’s not that quite good with other household stuff… I feel that I don’t want to lose my career. I love to work. I love to become a lecturer. And sadly, I have to leave that, I have to resign from that position… I really regret that.*”

—Student visa, 2004

For the most part, women’s journeys to Australia were mostly facilitated by other Indonesians, often currently living in Perth. These social networks often worked to reinforce cultural practices, including traditional gender roles and provided a sense of belonging for women. Social networks were a mixture of close and extended ties, often using bridging social capital. For example, women often knew of someone who had previously lived in Perth, who was then able to connect them to someone living in Perth currently. Women connected with these support people (via social media, predominately WhatsApp) prior to their arrival who then facilitated accommodation and schooling for children close to other Indonesians. Support people were willing to assist participants to settle into life in Perth and continued to be an ongoing source of support. Single women reported they often lived with other single Indonesian women. These social networks were important for women in forming a sense of community and connection in Perth. For women who had migrated with the support of other Indonesians, they maintained close ties with the Indonesian community, continuing to reinforce gender roles.

#### 3.1.3. ‘Moving Here Is So Liberating’: Challenging Traditional Gender Roles

In contrast, women who self-initiated migration often challenged traditional gender roles, typically migrating for employment or education opportunities or for ‘Western’ relationships. Moving to a Western country was seen as an opportunity for freedom or independence—for example, being able to have a career or to explore new ideas of sexuality. Participant 2’s decision to migrate to Perth, to “marry a Western man”, was driven by her wanting to have a career and the rejection of the traditional gender role of caretaker. Participant 7 moved to Australia for the opportunity to have “independence” as a single mother, characterised as having employment, financial independence, and “power”. She had been deterred from receiving an education by her parents, but in moving to Perth she challenged this gender norm and enrolled in university.

“*Yeah. I wanted to go to university. (Parents) say, “But you’re a girl.” You know, they still think that boys deserve more education than girls. … you get married and then, you know—just like that. But for boys, they’re willing to pay for their education… But then I think years after that, I can go to the university by myself.*”

—Student visa, 2018

### 3.2. Country of Destination: Being ‘Temporary’

#### 3.2.1. ‘You Can Disappear’: Temporary Visas Limiting Help Seeking and Reinforcing Gender Roles

Several women who were, or had been, on temporary visas in Australia expressed their feelings of being unsettled or not belonging in Australia. Their temporary visa status meant they were unable to apply for work, access social services, or access health services without cost. Temporary visas were not always ‘temporary’, with some women describing being on temporary visas for upwards of several years. Women reported being on several different visas over the years, to keep them in Australia, moving between tourist, student, and partner visas. Being on a temporary visa meant women were unable to work, often pushing women to be financially reliant on their male partners and without access to health and other services for years. One woman described this situation as “being a prisoner in this country,” in which she was only allowed to care for her husband and children. She described being unable to access health services without her partner driving and paying for the appointment.

Temporary visas made women feel uncertain, with concerns about being deported back to Indonesia. Women avoided causing “trouble”, which included not accessing health services or services such as the police. One woman described her experience of being sexually assaulted by a person in a position of power, and not reporting the incident due to beliefs that this would get the family deported from Australia. As such, she received no support after the assault (such as health services or counselling) and kept silent about the incident:

“*And then even when I got home that night there was like bruise marks on me where he grabbed me, my mum still didn’t call the cops. … everyone just like ‘Oh, you can disappear!’ and of course all Asians have heard horror stories from America. We don’t know if Australia is the same. Getting deported.*”

—Permanent resident, 2012

#### 3.2.2. ‘Good Girls’: Maintaining Gendered Expectations of Sexuality

When prompted about gender roles, women described “good girls”. “Good girls” described socially acceptable behaviour for Indonesian women, representing traditional gender norms. Maintaining “good girl” behaviour has nuanced implications for sexual health; while it encouraged monogamous sexual relationships, it also delayed help-seeking for sexual health issues due to shame or concerns about social ramifications. When prompted about what defined a “good girl”, women described someone who did not have sexual intercourse before marriage, was monogamous to her husband, dressed modestly, and was selfless in looking after her children and husband. Women also noted that “good girls” didn’t discuss sexual health, including with health professionals. Women noted there was not an equivalent term for men.

For women with Indonesian husbands, they reflected that living in Australia had not changed their relationships or sexual behaviour, and they continued to embody “good girl” practices. They described sex as a duty in marriage, and not to be discussed even with their husbands. Women were not expected to derive pleasure or initiate sex with their husbands or discuss safer sexual practices. In interviews, women commented that discussing sex was “so embarrassing” and was a deterrent to discussing sexual health with partners or a health provider. While several women had engaged with sexually explicit material prior to marriage, they had no knowledge about issues such as contraception or gynaecological health conditions and weren’t sure what constituted “being healthy down there” (i.e., what was considered normal vaginal health or what was normal during sexual intercourse). For most women, there was a strong reluctance to use words such as ‘sex’ or to use the name of female genital parts. For those who were able to use this language during the interviews, they often expressed that it was the first time they had done so:

“*So when I start to have that, um, to have sex and everything with my husband, that’s when I start learning things related to that sexual stuff. But luckily, I don’t have any problem with the healthy side, especially in my—that part (vaginal area). [laughs] See. You noticed me. I still cannot say that.*”

—Student visa, 2004

In maintaining “good girl” values, women held a pragmatic view of marriage. Women believed that if their husbands provided financially for them, they had to respect him as part of their marriage duty. It was accepted that men might be unfaithful to their wives, engaging in extra-marital relationships, but this was socially tolerated. This acceptance of a husband’s multiple sexual partners, coupled with a reluctance to discuss safer sexual practices and to access sexual health services, have important implications for the transmission and late diagnosis of STIs.

“*You know, Indonesia has a history with patriarchal culture where men become a leader of the family, the decision-maker, the breadwinner, and wives should respect the husband… But at some point, there’s some value that we still keep because of our religion as well because we have to respect our husband… as long as my husband still love me, still take care of my children, still responsible to the family, so, if he has lust (sexual intercourse) with somebody else, but put the wife more priority, well, I think, that’s okay*.”

—Student visa, 2016

Religion was an important part of participants’ identities and most women actively engaged in religious practices (i.e., prayer, attending services, and upholding values) that continued in Australia. However, once in Australia, women had to navigate between their faith and the transformation of gender norms and/or sexuality. In many cases, religion in part reinforced traditional gender norms, such as sex as a duty of marriage or the value of women’s virginity. Religion, connected to culture, emphasised women having to self-police their sexuality. This was especially the case when women attended services or groups with other Indonesians, where gender norms from Indonesia were often upheld in Australia.

#### 3.2.3. ‘They Talking behind me and How Bad I am’: How Anticipating Return Influences Sexuality While in Australia

Anticipation of returning to Indonesia (either permanently or temporarily) maintained gender norms and influenced women’s sexuality whilst in Australia. Women were concerned with how their behaviour, or attitudes, would be perceived by people living in Indonesia. This was experienced by both women who had permanent resident status in Australia and temporary visa holders. Women who had permanent resident status in Australia remained strongly connected to Indonesia. Women frequently returned to Indonesia for vacation, for business, for health, or to see family and friends (including sexual partners), with some women describing this as having “two homes”. Some women intended to live permanently in Indonesia after a certain length of time in Australia (such as retiring in Indonesia). Being ‘permanent’ in Australia appeared to facilitate more travel back to Indonesia for legal and financial factors. For this reason, women were cautious of maintaining “good girl” practices.

Women on temporary visas, who anticipated returning to Indonesia, described being wary of how their attitudes and behaviour in Australia may be perceived upon their return. “Gossip” through transnational social networks meant that women were still expected to adhere to Indonesian gender norms. Single women described concerns that gossip would reach its way back to their families in Indonesia, and the social consequences of this when they returned (i.e., the ability to marry).

For some women, time in Australia had shifted their own attitudes towards gender and sexuality. Women were supportive of Australian attitudes and policies around gender and sexuality (i.e., acceptance of sex before marriage, women owning properties, etc.), and weren’t sure how they would reconcile these differences once home in Indonesia. Women described anticipating missing things about Australia, like the ability to wear lipstick without fear of being seen as “hijab boobies” (where you wear a hijab, but your body is not respectful i.e., wearing lipstick).

### 3.3. Country of Destination: Two Homes

This section describes women’s experiences of belonging in Australia. It is important to note that all women identified as being Indonesian, despite living in Australia, and maintained a connection to their community and culture.

#### 3.3.1. ‘Being a Banana’: Becoming More Open-Minded about Sexuality

Some women felt that their time in Australia had made them more ‘Western’. Women used the expression of being a “banana” to describe their Asian appearance (‘yellow’), but their sense of connection or belonging to a more ‘Western’ culture (white). This notion of becoming ‘Western’ was difficult to explain but included becoming more ‘open-minded’ about sexuality (i.e., not concerned with sex before marriage, same-sex relationships), embracing new gender norms and roles (i.e., women’s education and employment after marriage), a preference for particular foods, and having good English and an understanding of jokes and slang:

“*So yeah, and like moving here everything is so liberated and I’ve never really explored sexually and learned proper sexual health stuff until I moved here.*”

—Permanent resident, 2012

Women considered both Indonesia and Australia to be home, with some women frequently living between both countries. Women maintained their connections to Indonesia through social media interactions with friends and family living in Indonesia, through reading the daily Indonesian news, and by voting in Indonesian elections. However, they often separated themselves from the Indonesian community in Perth, citing differences in sexuality and gender norms between Australia and Indonesia which made it difficult to connect with other women.

#### 3.3.2. ‘White Knights’: Relationships with Australians Supporting Changing Gender Roles

More than half of the women interviewed either had an Australian partner or were planning on meeting one. Women with Australian partners retold their family’s excitement that they had married a “white foreigner”. This was seen as having a higher status in Indonesia and was associated with wealth; women described other Indonesians as having “more respect” for their husbands. Women who planned on finding an Australian husband spoke about the freedom that it would provide and the opportunity to have employment. They spoke about Indonesian men expecting women to be “housewives only”, however some women wanted to have careers and felt that this aspiration would not be supported by an Indonesian man:

“*Because for me, I think, if we are married, for one, [Indonesian husband] like take away our freedom. I don’t want to live like that.*”

—Student visa, 2019

Several women had relationships mostly with Australians; predominately women who had an Australian (white) partner. For women who weren’t married, they described Australian friends being accepting of sex before marriage and supportive of discussing sexual health. Australians facilitated women to explore their sexuality and reshape gender norms, while Indonesian networks were seen as less receptive towards sex. As such, women who self-reported having more Australian friends described being more proactive about safer sexual practices, supported by their social networks:

“*Australians are super chilled and friendly and open about things. If I were to talk about things with Asian Indonesians mostly here, they tend to be a bit almost innocent like, close-minded innocent like. I would mention about the things (sexual behaviour)… and they’d look at me as if I’ve committed a great taboo. Yeah, so that’s why I’m not really close to Indonesians here.*”

—Permanent resident, 2012

#### 3.3.3. ‘Open-Minded’: Being in Australia, Freedom of Sexuality and Sexual Health Risk

Arriving in Australia provided new opportunities for women to openly explore their sexuality. They did this by taking part in discussions about sex with Australian-based friends, participating in social media, and engaging in new relationships (particularly with people not from Indonesia). Australia was viewed as being ‘open-minded’ or liberal—women used examples of sexual health education in schools, sexualised media, legal acceptance of de-facto and same-sex relationships, acceptance of casual sexual relationships, and public displays of intimacy and affection (i.e., hand holding, kissing). Women’s exposure to Australia’s more liberal approach to sexuality (such as same-sex marriage and social acceptance of sex before marriage), challenged women’s idea of what was morally good or bad. For example, while one participant strongly identified as a Christian, she felt her decision to have sex before marriage, rather than “saving oneself”, did not affect her relationship with God. She no longer considered this behaviour as being a sin. However, for another woman, there existed tension in her decision to divorce her husband as “it is a sin” and “God hates people who divorce.”

As women engaged in sex before marriage, they were exposed to new sexual risks. With limited sexual health literacy pre-migration, women relied predominately on Australian males to help negotiate contraception. They described how males emphasised concerns about pregnancy, had little concern for STIs, and disliked condoms. Women, too, were mostly concerned about pregnancy and its implications. Having a child whilst unmarried was not perceived to be socially acceptable in the Indonesian community. For one woman, her unplanned pregnancy was the predominant reason for marrying her Australian partner, despite not living in Indonesia. While STIs were considered easy to treat (particularly in Australia) and not visible, pregnancy, had very real implications for women’s ability to study, find employment, and continue their careers. Sexual health testing for women was often only undertaken when prompted by a male partner.

“*When you have a kid, you have no freedom… when we have kid, we will lose our future. We cannot go finish our study. We cannot go working... But then with, you know, health issue (STI)… If you take your medicine and after then go check up in a doctor and after medicine, it doesn’t really like, affect your future, kind of thing? But then if you’re pregnant, it’s kinda like my future is ruined... there is always, always a moral judgment like people judge you and whatever, but then you can actually still, as a female, you can apply job and then they wouldn’t ask you for that (STI results). You get a job functioning as a human being, normal human being according to them. But then if you’re a pregnant woman? No. The doors close. At all. So you have no future.*”

—Permanent resident, 2007

## 4. Discussion

Limited literature exists on migration, gender, and sexuality; what has been written focuses predominately on migrants’ experiences post-migration [2] or solely describes limited sexual health knowledge pre-migration [47]. This research provides some insight into how the migration process, including anticipation of return, shapes women’s sexuality. Circumstances of migration were often dependent on gender and worked to either reshape or reaffirm traditional gender roles whilst in Australia. Expectations of return saw women ‘self-police’ sexuality and maintain traditional gender roles in anticipation of how their time in Australia would be viewed by those living in Indonesia. The migration journey and subsequent reshaping or maintenance of gender norms had an impact on women’s sexuality, including sexual health risks and access to sexual health services.

Similar to findings relating to sexual agency amongst married migrant and refugee women by Hawey et al. (2019), migration either maintained or challenged patriarchal views of women’s sexuality and gender norms. In this research, migration that was male-led generally reinforced traditional gender roles pre- and post-migration. Pre-migration, women’s role as a ‘caretaker’ saw men have authority over decisions of when, and where, migration occurred, sometimes at expense of women’s careers or education in Indonesia. Migration to a more ‘liberal’ country like Australia, where there are fewer laws discriminating against gender, did not translate to increased power and decision-making for women. Instead, migration policies and processes often placed women as ‘dependent’ on males, undermining women’s power, consistent with what has been reported elsewhere [17]. In doing so, women became more reliant on men for finances and for visa requirements. Additionally, and perhaps related to their dependency on husbands, women who migrated with men maintained traditional expectations around marriage. Women treated sexual intercourse as a duty, prioritizing their husband’s pleasure, which extended to the social acceptance of men engaging in extra-marital sexual affairs. Women maintained expectations of being sexually naïve, including being unable to name genitals (in any language), and describing their own reluctance to discuss sexual health—even with a health professional. Financial dependency and reliance on husbands for visas, coupled with maintenance of gender norms and cultural modesty, may in part explain lower rates of screening, such as for cervical cancer and breast cancer [23]. There are also implications here for the transmission of STIs and subsequent late diagnosis amongst women. Reviewing existing migration policies and procedures, and the effect on women’s status may improve health outcomes for women [48]. In the interim, other studies have posited the need for further work with men, to achieve gender and sexual health equity [49].

In examples of migration led by women, this was often done in the absence of men, and often in attempts to reshape traditional gender norms. Some women migrated to find new ‘Western’ relationships, supportive of education and employment opportunities. Australia was described as ‘open-minded’, where it was socially acceptable for women not to be married, to have sex outside of marriage, and for same-sex relationships. Australian men were seen to be more accepting of women having careers, thus providing more financial independence. Migration to Australia enabled women to explore their sexuality; however, women had limited sexual health literacy, putting them at risk of unintended and unwanted sexual health outcomes. Women relied on male sexual partners to help navigate contraception, which often emphasized pregnancy prevention without STI protection (i.e., the oral contraceptive). As reported elsewhere [50], women in this study reported sharing concerns about unplanned pregnancy and subsequent ‘shame’ associated with this. Unplanned pregnancy was described in relation to the impact on social status (both theirs and their families) and the negative impact on careers in becoming primary caregivers. Consistent with other literature [2,47], women in this study had limited knowledge of other STIs and modes of preventing STIs. Sexual health testing was only undertaken when prompted by an Australian male partner and male partners were perceived to hold power in both negotiating contraception use and in sexual health testing. While sexual health education upon resettlement is often recommended [51,52,53], our findings also suggest a greater emphasis on encouraging condom usage and sexual health testing amongst men (including Australian-born) in parallel.

Previous literature has criticized the homogenous grouping of “migrants”, without acknowledging differences between visa statuses and circumstances, and how these change over time [1]. Visa status has important implications for health service access in destination countries, broader structural factors such as employment and housing [11], as well as impacting the likelihood of return to their home country. Previous studies exploring migrant sexual health have mostly included permanent migrants; with a lack of attention on temporary residents (including international students) rendering their health and well-being “invisible to policy makers” (p. 151) [54]. In this study, we considered the ways in which women felt ‘temporary’ in Australia. This was not always related to visa status; though concerns about being deported or not being entitled to services (such as police or health) were mostly unique to temporary residents, and reported elsewhere [32]. Some temporary migrants held expectations that they would live permanently in Australia and moved between visas (or returned home) as needed to keep them connected to Australia. Women engaged in intimate relationships with Australian men and had children, whilst being on ‘temporary’ visas, with fears that they wouldn’t be able to stay. Limited literature exists on the experiences of ‘long-term temporary’ residents [55], and subsequent impacts on the health and well-being of individuals, particularly women. Conversely, women on permanent visas maintained strong, transnational ties to Indonesia, as reported in our previous work. Women on permanent visas upheld Indonesian community expectations of ‘good girl’ behaviour and had expectations of returning to Indonesia. This indicates that, while visa status gives some insight into some of the structural factors that influence migrant health, there is utility in understanding how expectations of remaining or returning, may affect behaviour and subsequent health outcomes. This work also further positions migration as a determinant of health, and how structural inequalities work to undermine health [11,12]. Additionally, there may be merit in public health interventions that exist in both countries and provide support for women throughout the migration journey, considering the utility of transnational social support.

### Strengths and Limitations

There are several limitations to this study. Interviews were conducted in English, without the option for an interpreter; experiences of women with limited English may be different. Women were predominantly married with children and on temporary visas, however, this is consistent with demographic data of Indonesian women living in Perth [56]. This research was exploratory and did not intend to be generalizable. Additionally, this research was conducted while all participants were living in Australia, capturing only how anticipation of return influenced health. There would be merit in further research exploring how a return to Indonesia does or does not influence gender roles and norms and potential health impacts.

We experienced difficulties in recruiting unmarried women, which may be due to the community-based methodology of this project and sex being considered a taboo subject, particularly among unmarried women. Though data were intended to be co-analysed, the close ties between Indonesian women and the private nature of the discussions made it challenging to share transcripts from interviews with community researchers. To manage this, the researcher broadly described emerging themes with community researchers and further refined them with the academic team. Being a cultural outsider, it is likely that the researcher has made assumptions about Indonesian culture, or “Westernised” concepts—this may have been mitigated by the researcher’s close connection with community researchers throughout the research. While a cultural outsider, the researcher may be considered an ‘insider’ given her strong connections to the Indonesian community researchers and participants—this may have deterred women from sharing their experiences.

A community-based approach is a strength of this project. The researcher had previous contact with most participants, which may have enhanced trust in the researcher and their credibility to conduct the research, potentially eliciting more detailed responses from participants. Interviews were informal and unstructured, and of considerable length—this allowed the researcher to develop rapport with participants and better explore women’s experiences.

The use of interviews after focus groups provided women with the opportunity to reflect on what was shared in a group setting and prompted discussions on what was agreed or disagreed with. While it is beyond the scope of this article, we reflect that women sometimes disagreed with or contradicted what was initially said in focus groups—perhaps suggesting the influential role of social desirability bias in close communities.

## 5. Conclusions

Our findings suggest that migration influences, and is influenced by, gender, and may work to reaffirm or challenge traditional gender norms and roles for migrant women. We found that a temporary visa status often reaffirmed traditional gender roles, placing women dependent on men, with implications for sexual health. Migration policies and procedures, and their impact on women, should be reviewed. We found that circumstances of migration and anticipation of return influenced sexual health attitudes and behaviour. Women maintained gendered norms where migration was male-led or return to Indonesia was anticipated. Women-led migration enabled new intimate relationships, but often in the context of limited sexual health literacy. Findings indicate that greater consideration of migration, alongside visa status, is needed in public health interventions. For example, interventions that utilize ongoing social networks between countries. More attention is needed on addressing the structural and sociocultural factors of migration that impact sexual health outcomes. 

## Figures and Tables

**Table 1:** Demographic data for interview participants (demographic data missing for one participant).

Year of Birth *	Number of Women
1990–1999	1
1980–1989	5
1960–1979	2
Religion	
Islam	3
Christian	3
Hindu	1
Buddhist	1
Catholic	1
Language(s) spoken	
Indonesian	9
Javanese	1
Sundanese	1
Balinese	1
Mandarin	1
Self-reported English Proficiency	
Very well	4
Well	4
Not well	1
Year of arrival in Australia	
2015–2018	6
2010–2014	2
2004–2009	1
Status in Australia	
Permanent resident	3
Student visa	5
Tourist visa	1
Education	
Masters	2
University degree	5
Year 12	1
Some high school	1
Relationship Status	
Married	7
Unmarried	1
In a relationship	1
Children	
Yes	7
No	2
HIV test	
Yes	4
No	5

* Age missing for one participant.

**Table 2:** Themes and sub-themes.

Themes	Sub-Themes
Pre-departure and travel: motivations, support, and gender roles	‘I’m a little curious’: expectations of marriage, sexual naivety, and self-taught sexual education
	‘You already know the culture’: maintaining gender roles in migration
	‘Moving here is so liberating’: challenging traditional gender roles
Country of destination: being ‘Temporary’	‘You can disappear’: temporary visas limiting help seeking and reinforcing gender roles
	‘Good girls’: maintaining gendered expectations of sexuality
	‘They talking behind me and how bad I am’: how anticipating return influences sexuality while in Australia
Country of destination: two homes	‘Being a Banana’: becoming more open-minded about sexuality
	‘White knights’: relationships with Australians supporting changing gender roles
	‘Open-minded’: being in Australia, freedom of sexuality and sexual health risk

## Data Availability

The data presented in this study are available on request from the corresponding author. The data are not publicly available due to ethical reasons.
